# Statement from the frontal fibrosing alopecia international expert alliance: SOFFIA 2024

**DOI:** 10.1111/jdv.20833

**Published:** 2025-07-23

**Authors:** Nekma Meah, Jane Li, Dmitri Wall, Katherine York, Bevin Bhoyrul, Laita Bokhari, Lachlan Coulthard, Leila Asfour, Leonardo Spagnol Abraham, Daniel Asz‐Sigall, Wilma F. Bergfeld, Regina C. Betz, Ulrike Blume‐Peytavi, Valerie Callender, Vijaya Chitreddy, Andrea Combalia, George Cotsarelis, Brittany Craiglow, Rachita Dhurat, Ncoza Dlova, Jeff Donovan, Andrei Doroshkevich, Samantha Eisman, Paul Farrant, Aida Gadzhigoroeva, Jack Green, Ramon Grimalt, Matthew Harries, Maria Hordinsky, Alan D. Irvine, Victoria Jolliffe, Spartak Kaiumov, Brett King, Steven Kossard, Joyce Lee, Won‐Soo Lee, Nino Lortkipanidze, Amy McMichael, Natasha Atanaskova Mesinkovska, Andrew Messenger, Paradi Mirmirani, Elise Olsen, Seth J. Orlow, Yuliya Ovcharenko, Bianca Maria Piraccini, Rodrigo Pirmez, Adriana Rakowska, Pascal Reygagne, Janet Roberts, Lidia Rudnicka, David Saceda‐Corralo, Jerry Shapiro, Pooja Sharma, Tatiana Silyuk, Poonkiat Suchonwanit, Anita Takwale, Antonella Tosti, W. I. Visser, Sergio Vañó‐Galván, Annika Vogt, Martin Wade, Leona Yip, Abraham Zlotogorski, Cheng Zhou, Rodney Sinclair

**Affiliations:** ^1^ Mersey and West Lancashire Teaching Hospitals NHS Trust St Helens UK; ^2^ Manchester University, Faculty of Biology, Medicine and Health Manchester UK; ^3^ Department of Medicine (Dermatology) St Vincent's Hospital Melbourne, the University of Melbourne Fitzroy Victoria Australia; ^4^ The Skin Health Institute Carlton Victoria Australia; ^5^ Department of Dermatology, Box Hill Hospital Eastern Health Box Hill Victoria Australia; ^6^ Hair Restoration Blackrock Dublin Ireland; ^7^ National and International Skin Registry Solutions (NISR), Charles Institute of Dermatology University College Dublin Dublin Ireland; ^8^ The Mater Misericordiae University Hospital Dublin Ireland; ^9^ Beachside Specialist Group Noosa Heads Australia; ^10^ Sinclair Dermatology Melbourne Australia; ^11^ Salford Royal Foundation Trust, Northern Care Alliance Manchester UK; ^12^ Trichology Unit Hospital Regional da Asa Norte Brasilia Brazil; ^13^ Programa de Pós Graduação Em Ciências Médicas (PPGCM) Universidade de Brasília (UnB) Brasilia Brazil; ^14^ Trichology Clinic, Dermatology Department Dr Manuel Gea Gonzalez General Hospital Mexico City Mexico; ^15^ Cleveland Clinic Main Campus Cleveland Ohio USA; ^16^ Institute of Human Genetics University of Bonn, Medical Faculty & University Hospital Bonn Bonn Germany; ^17^ Department of Dermatology, Venerology and Allergology, Clinical Research Center for Hair and Skin Science Charité‐Universitaetsmedizin Berlin Berlin Germany; ^18^ Howard University College of Medicine Washington, DC USA; ^19^ Callender Dermatology & Cosmetic Center Glenn Dale Maryland USA; ^20^ Dermatology Department Hospital Clinic de Barcelona Barcelona Spain; ^21^ Perelman School of Medicine at the University of Pennsylvania Philadelphia Pennsylvania USA; ^22^ Yale Department of Dermatology New Haven Connecticut USA; ^23^ Department of Dermatology LTM Medical College & Hospital Sion Mumbai India; ^24^ Chief Specialist and HOD Department of Dermatology NRMSOM, UKZN Durban South Africa; ^25^ Dean and Head School of Clinical Medicine University of KwaZulu Natal Durban South Africa; ^26^ Department of Dermatology University of British Columbia Vancouver British Columbia Canada; ^27^ Donovan Hair Clinic Whistler British Columbia Canada; ^28^ Hair Treatment and Transplantation Center Saint Petersburg Russian Federation; ^29^ Brighton and Sussex University Hospitals NHS Trust Brighton UK; ^30^ Scientific & Practical Center Dermatolovenereology and Cosmetology of the Moscow City Health Department Moscow Russian Federation; ^31^ Institute of Beautiful Hairs Moscow Russia; ^32^ St.Vincent's Hospital, Skin Health Institute Melbourne Victoria Australia; ^33^ Facultat de Medicina i Ciències de la Salut Universitat Internacional de Catalunya Sant Cugat del Vallès (Barcelona) Spain; ^34^ Salford Royal Hospital, Northern Care Alliance NHS Foundation Trust, Manchester Academic Health Science Centre Manchester UK; ^35^ Centre for Dermatology Research, Faculty of Biology, Medicine and Health University of Manchester & NIHR Biomedical Research Centre Manchester UK; ^36^ Department of Dermatology University of Minnesota Minneapolis Minnesota USA; ^37^ Clinical Medicine Trinity College Dublin Dublin 2 Ireland; ^38^ Centre for Cell Biology and Cutaneous Research Blizard Institute London UK; ^39^ Nautilus Clinic and Education Centre Saint Petersburg Russian Federation; ^40^ Yale School of Medicine New Haven Connecticut USA; ^41^ Director Kossard/ Laverty Dermatopathologists Macquarie Park New South Wales Australia; ^42^ National Skin Centre Singapore; ^43^ Department of Dermatology Yonsei Wonju University Wonju Korea; ^44^ David Tvildiani Medical University Head of Department of Dermatology and Venereology Tbilisi Georgia; ^45^ Department of Dermatology Wake Forest School of Medicine Winston‐Salem North Carolina USA; ^46^ Department of Dermatology University of California, Irvine Irvine California USA; ^47^ University of Sheffield Sheffield UK; ^48^ Kaiser Permanente Vallejo, Department of Dermatology Vallejo California USA; ^49^ Duke Dermatology Clinic Durham North Carolina USA; ^50^ The Ronald O. Perelman Department of Dermatology New York University School of Medicine New York New York USA; ^51^ Department of Pediatrics New York University Medical Center New York New York USA; ^52^ Department of Infectious Diseases and Clinical Immunology of the V.N. Karazin KharkivNational University Kharkiv Ukraine; ^53^ Dermatology Unit IRCCS Azienda Ospedaliero‐Universitaria di Bologna Bologna Italy; ^54^ Department of Medical and Surgical Sciences University of Bologna Bologna Italy; ^55^ Instituto de Dermatologia Professor Rubem David Azulay, Santa Casa da Misericórdia Do Rio de Janeiro Rio de Janeiro Brazil; ^56^ Department of Dermatology Medical University of Warsaw Warsaw Poland; ^57^ Directeur du Centre de santé Sabouraud Hopital Saint Louis Paris France; ^58^ Northwest Dermatology Institute Portland Oregon USA; ^59^ Dermatology Department Hospital Universitario Ramón y Cajal Madrid Spain; ^60^ IRYCIS, Universidad de Alcalá Madrid Spain; ^61^ Division of Dermatology, Department of Medicine, Faculty of Medicine, Ramathibodi Hospital Mahidol University Bangkok Thailand; ^62^ Department of Dermatology Gloucestershire Hospitals NHS Foundation Trust UK; ^63^ Dr. Phillip Frost Department of Dermatology and Cutaneous Surgery University of Miami, Miller School of Medicine Coral Gables Florida USA; ^64^ Division of Dermatology, Department of Medicine, Faculty of Medicine and Health Sciences Stellenbosch University Cape Town South Africa; ^65^ Director of Hair Disorders Unit and #TricoHRC Research Group Madrid Spain; ^66^ The London Skin and Hair Clinic London UK; ^67^ Skin Partners Specialist Dermatologists Brisbane Australia; ^68^ Department of Dermatology, Hadassah Medical Center Hebrew University of Jerusalem, the Faculty of Medicine Jerusalem Israel; ^69^ Department of Dermatology Peking University People's Hospital Beijing China; ^70^ Department of Medicine University of Melbourne Melbourne Victoria Australia

**Keywords:** consensus, Delphi, frontal fibrosing alopecia, therapeutic guideline

## Abstract

**Background:**

As the incidence of frontal fibrosing alopecia (FFA) continues to rise, there is a need for an optimal treatment algorithm for FFA.

**Objectives:**

To produce an international consensus statement on the treatment modalities and prognostic indicators of FFA.

**Methods:**

Sixty‐nine hair experts from six continents were invited to participate in a three‐round Delphi process. The final stage was held as a virtual meeting facilitated via Zoom. The consensus threshold was set at ≥66%.

**Results:**

Of 365 questions, expert consensus was achieved in 204 (56%) questions following completion of the three rounds. Three additional questions were included at the final meeting. The category with the strongest consensus agreement was disease monitoring (9; 100%). Questions pertaining to physical therapies achieved the least category consensus (15; 40%), followed by systemic therapy (45; 43%).

**Limitations:**

The study lacked sufficient representation from Africa and South America.

**Conclusions:**

SOFFIA highlights areas of agreement and disagreement among experts. Robust research is warranted to provide evidence‐based treatment recommendations.

## Graphical abstract

Optimal treatment for frontal fibrosing alopecia (FFA) remains contentious. An international expert panel agreed that highly/ultra‐potent topical steroids were the preferred first‐line topical therapy. Preferred systemic therapies were 5‐alpha reductase inhibitors, followed by hydroxychloroquine. Low‐dose isotretinoin was the preferred treatment for facial papules, and intralesional corticosteroids for FFA‐induced eyebrow alopecia.

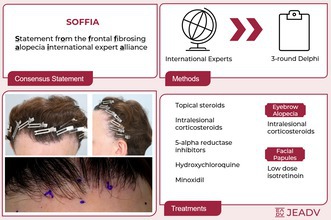

**Why was the study undertaken?**

To address the need for an optimal treatment algorithm for FFA.

**What does this study add?**

This is the first large‐scale international consensus on the treatment modalities and prognostic indicators of FFA.

**What are the implications of this study for disease understanding and/or clinical care?**

This international consensus identifies therapeutic strategies based on expert experience, but also the need for more effective, evidence‐based treatments for FFA.

## INTRODUCTION

**FIGURE 1:**
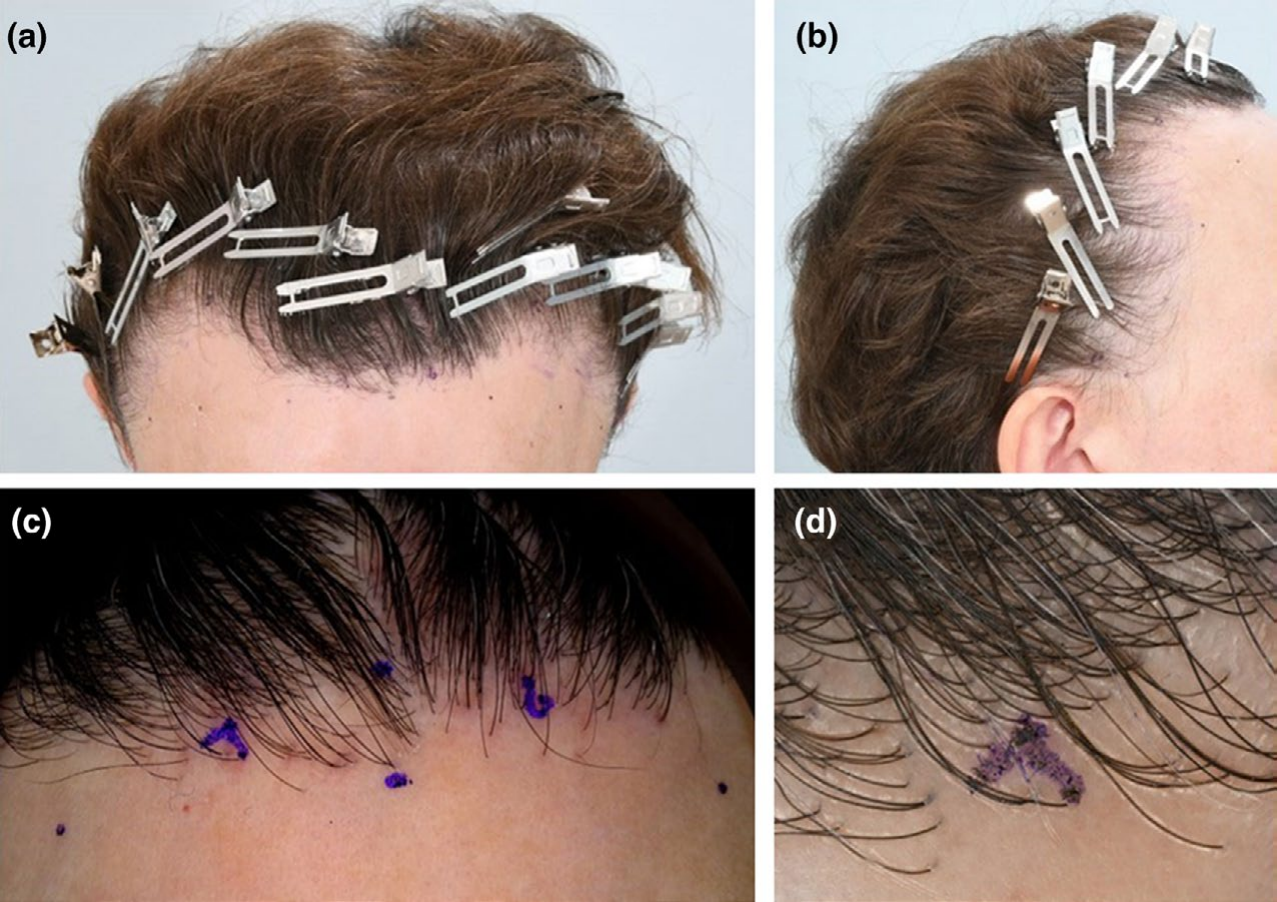
(a–c) Series of photos from a patient with frontal fibrosing alopecia (FFA). (a, b) Demonstrate frontotemporal recession. (c) Magnified view of the frontal hairline utilizing a TrichoLAB research tool showing perifollicular erythema, scale, single hairs, patchy hair loss and absence of vellus hair. (d) Trichoscopy shows single hairs, perifollicular scale and subtle perifollicular erythema.

Frontal fibrosing alopecia (FFA) is a form of lymphocytic primary cicatricial alopecia. Following the first reports in 1994,^1^ its incidence has risen rapidly,^2^ so much so that it is now regarded as the most common type of primary cicatricial alopecia (Figure 1a–d). Despite considerable efforts to determine the aetiology, clinicopathological characteristics and best therapeutic approach, guidelines (national or international) are lacking. For the general dermatologist, management of this hair loss condition can be daunting. For this reason, there is a clear need for guidance on the treatment of FFA by hair experts.

This paper describes international consensus findings on treatments, parameters for disease monitoring and prognostic indicators for FFA from hair experts. While there remains a clear need for robust treatments for FFA,^3^ management can undoubtedly be challenging. Herein we provide guidance regarding existing and emerging therapies.^4^

## METHODS

### Expert panel selection

Sixty‐nine dermatologists with recognized expertise in hair and scalp disorders were invited to participate in the **s**tatement fr**o**m the **f**rontal **f**ibrosing **a**lopecia **i**nternational expert **a**lliance **(**SOFFIA). Most experts had contributed to previous alopecia‐related Delphis. Individual recommendations from colleagues regarding expertise in FFA were also considered. Importantly, global diversity and representation from the International Federation of Hair Research Societies (IFHRS) were prioritized.

### Delphi survey

FFA was identified as a Delphi topic from an expert group poll. Experts submitted FFA treatment and prognosis‐related questions that contributed to a preliminary questionnaire created by a core panel of six dermatologists (JL, NM, DW, BB, KY and RS) and one research scientist. Two further expert panels undertook further revisions.

### Delphi process

The Delphi process facilitates the development of numerous core outcome and diagnostic criteria^5^, ^6^, ^7^, ^8^, ^9^, ^10^ and is ideal where representation is required from geographically disparate participants.

Convergence of opinion through iterative rounds is facilitated anonymously, as each round enables revision of responses in the context of non‐identifiable group voting.

SOFFIA involved two questionnaire rounds followed by a final Zoom meeting.^11^ Participants were instructed that questions pertained to female FFA patients. All questions utilized a five‐point Likert scale (1 = strongly disagree; 2 = disagree; 3 = neither agree nor disagree; 4 = agree; 5 = strongly agree; or ‘not applicable’). For Round 3 only, a three‐point scale (‘agree’, ‘disagree’ or ‘neither agree nor disagree’) was adopted. Participants were permitted to comment for all rounds. Anonymized comments for each question were viewable by all participants for all rounds and enabled submission of additional questions for Round 3 discussion. Live voting at the third round was permitted using PollEV software.^12^

### Consensus threshold

Consensus threshold, based on prior eDelphi precedence, was set at ≥66% participant agreement (scores 4–5) or disagreement (scores 1–2), while a score of 3 was considered indeterminate. The consensus threshold for SOFFIA was selected taking into account the contentiousness of FFA among experts.

Questions achieving consensus or considered low probability of achieving consensus (≤33%) were excluded from the next round.

## RESULTS

### Expert panel

Of the 69 FFA experts invited, 61 (88%) completed Round 1, 58 (84%) Round 2 and 42 (61%) attended the virtual Zoom meeting on 11 December 2020. Twenty‐six (43%) work in public (+/−academic institutions) and private practice, 17 (28%) exclusively in private and 14 (23%) exclusively in public practice. Participants were from Europe (27; 44%), Asia (5; 8%), Australia (6; 10%), North America (16; 26%), South America (2; 3%) and Africa (3; 5%). Two experts did not state their country of practice. Years of clinical experience were not captured.

### Delphi rounds

**FIGURE 2:**
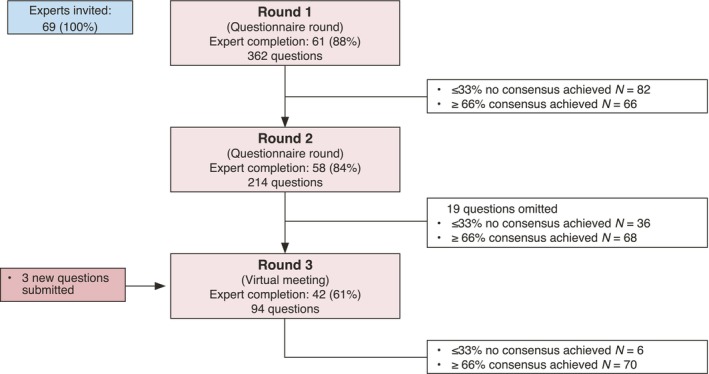
Summary of results from all Delphi rounds: SOFFIA.

Figure 2 summarizes the SOFFIA Delphi process. Overall, 365 questions related to treatment modalities, disease monitoring and prognostic indicators for FFA. Expert consensus was achieved in 204 questions (56%); with 66 questions achieving consensus after Round 1, 68 after Round 2 and 70 after Round 3 (Table S1). Three additional questions were submitted for Round 3.

Three hundred and forty‐six questions related specifically to FFA treatment: topical therapy, intralesional treatment, systemic therapies, physical therapies and initiation, discontinuation and usefulness of therapy. Table S2 summarizes the level of evidence for treatments (OCEBM criteria).^13^

Questions regarding regrowth were included. ‘Regrowth’ in the context of FFA refers to either viability of the inflamed FFA hair follicle or regrowth of other hairs, for example, co‐existing androgenetic alopecia.^14^, ^15^

## CONSENSUS OUTCOMES

Key: CA—consensus agreement; CD—consensus disagreement; NC—no consensus; AC—approaching consensus. Statements close to consensus termed: approaching consensus (AC) from Round 3 were also included, defined as * ≥ 50% <60%, ** ≥ 60% < 66%.

### Topical and intralesional therapies

Expert consensus for topical and intralesional treatments is summarized in Table 1.

**TABLE 1:** Expert consensus for topical and intralesional treatments in FFA.

Treatment	Expert consensus was achieved in the following
Topical corticosteroids	Topical steroids are effective for symptom control and in halting disease progression. They do not promote regrowth. Daily frequency is the optimal usage and trialled for at least 3–6 months, not greater than 6 months. The optimal strength (for anterior hairline involvement) is high potency/ultra‐potent not low/mid‐potency. Topical steroids can be ceased when the patient is asymptomatic together with resolution of perifollicular erythema/scale and hair pull test negative or, when the hairline is determined to be stable together with resolution of perifollicular erythema/scale. Preferably, topical steroid use should not be ceased if perifollicular erythema/scale is present even if the scalp is asymptomatic and hair pull test negative, and the hairline is determined to be stable. Ideally topical steroids should be weaned prior to cessation.
Topical calcineurin inhibitors	Topical calcineurin inhibitors as a group are effective for symptom control and in halting disease progression. They do not promote regrowth. Tacrolimus is an effective treatment option for FFA with an optimal therapeutic strength of 0.1% not 0.03%.
Topical minoxidil	Topical minoxidil is not effective to halt progression/ induce remission but may be effective in promoting regrowth. Topical minoxidil can be applied to the whole scalp or the involved scalp margin. Topical minoxidil can be applied to eyebrows and has an optimal strength of 5% not 2%.
Topical prostaglandins	Topical bimatoprost 0.03% or topical latanoprost 0.005% can be applied to the eyebrows. Topical bimatoprost 0.03% or topical latanoprost 0.005% is not an effective treatment option for scalp FFA and should not be applied to the whole scalp, nor the involved scalp margin.
Topical miscellaneous	Topical tretinoin, topical 1% dithranol (anthralin) or topical antihistamines are generally not effective treatment options for FFA.
Intralesional Therapy	Intralesional corticosteroid is an effective treatment option for FFA of the scalp/ FFA of the eyebrows in initial stage disease not late‐stage disease. Intralesional corticosteroids should be considered in patients with clinical signs of activity (e.g. peripilar scaling/ erythema) +/− presence of positive hair pull test. Intralesional corticosteroids should be considered in patients with clinical signs of activity in patients with progressive hair loss (e.g. on serial photography), even in the absence of visible scalp inflammation. For use on the scalp: intralesional corticosteroids have an optimal dose of 5 mg/mL not 10 mg/mL. For use on the eyebrows: intralesional corticosteroids have an optimal dose of 2.5 mg/mL not 10 mg/mL. The recommended interval between injections to the scalp or eyebrows is 6–8 weekly. Intralesional corticosteroid treatment can favourably influence FFA prognosis.

### Systemic therapies

One hundred and four questions were related to systemic treatment. Expert consensus was reached in 45 (43%).

#### Immune modulation

##### Hydroxychloroquine (HCQ)

Hydroxychloroquine is effective for symptom control and halting disease progression but does not promote hair regrowth. It is optimally dosed at 5 mg/kg/day^16^ and indicated in patients with moderate–severe disease (Physician Global Assessment), clinical signs of activity (e.g. peripilar scaling/ erythema), irrespective of a positive hair pull test. It is also indicated in progressive hair loss (e.g. on serial photography) even in the absence of visible scalp inflammation. It is not indicated in all patients routinely.

##### Systemic corticosteroids

Systemic corticosteroids are an effective treatment option for FFA, but use should be limited.

The optimal dosing of prednisolone equivalent was not determined. However, there was expert agreement that it was not <0.25 mg/kg daily nor 1 mg/kg daily. AC for 0.5 mg/kg daily*.

The duration of treatment with systemic corticosteroids before weaning was not determined; however, there was agreement that it should not be before 1 or 2 weeks.

##### Antibiotics

Doxycycline 100 mg once daily or mino cycline 50 mg twice daily are effective treatment option.

##### Antihistamines

Oral antihistamines are generally not an effective treatment option for FFA.

##### Naltrexone

There was no consensus for naltrexone effectiveness and dosing.

#### Steroid sparing therapies

Steroid sparing therapies are considered in patients with active progressive disease.

##### Ciclosporin

Ciclosporin is an effective treatment option for FFA and is optimally dosed at 3 mg/kg/day.

##### Methotrexate (MTX)

There was no consensus for MTX as an effective treatment option. If prescribed, optimal dosing of 15 mg weekly was agreed, not 25 mg nor 30 mg weekly (AC 10 mg**, 20 mg* weekly).

##### Mycophenolate mofetil (MMF)

There was no consensus for MMF as an effective treatment option. If prescribed, it should not be dosed below 1 g/day (AC 2–3 g/day*).

##### Janus kinase inhibitors (ruxolitinib, tofacitinib)

There was no consensus for JAKi effectiveness and dosing.

#### Hormonal modulation

##### 5‐alpha reductase inhibitors

5‐alpha reductase inhibitors (5ARIs) are effective treatments for FFA, regardless of co‐existent androgenetic alopecia. They halt disease progression/induce remission but do not promote hair regrowth. 5ARI may be considered in patients with clinical signs of activity (e.g. peripilar scaling/ erythema). They may be considered in patients with progressive hair loss (e.g. on serial photography) even in the absence of visible scalp inflammation. 5ARI should not be reserved only for FFA patients with concomitant androgenetic alopecia. Finasteride is optimally dosed at 2.5 mg daily, not 1, 1.25, 2, 3 and 4 mg daily. (AC 5 mg* daily). Dutasteride is optimally dosed at 0.5 mg daily, not 0.5 mg weekly.

##### Flutamide, Bicalutamide, Spironolactone

There was no consensus for effectiveness and dosing.

#### Others

##### Retinoids

Isotretinoin is an effective treatment option. The optimal dosing is 0.25 mg/kg/day, not 0.5 nor 1 mg/ kg/day (AC <0.25 mg/kg/day*). There was no consensus for acitretin effectiveness and dosing.

##### Pioglitazone

There was no consensus for pioglitazone's effectiveness and dosing.

##### Minoxidil

Oral minoxidil is an effective treatment option. It is most effective as an adjunct when used in combination with other treatments.

### Physical therapies

Thirty‐eight questions related to physical therapies. Expert consensus was reached in 15 (40%).

#### Surgical

##### Hair transplantation (HT)

Hair transplantation can be considered in patients with FFA if the disease is inactive. The optimal timing is after ≥24 months of inactivity. There was consensus disagreement for inactivity of 6 months duration (AC after 12 months of inactivity**). It can be considered in the absence of symptoms, perifollicular erythema/ follicular hyperkeratosis and positive hair pull test. Hair transplantation should not be considered based on the absence of progressive hair loss on serial photography alone, regardless of other signs. It should not be considered if signs of activity are present, even if performed while continuing medical therapy. HT may reactivate disease, and patients should be counselled accordingly. Typically, HT results in loss of >50% of the transplanted hairs >5 years post‐operatively* (AC). FFA HT patients should be maintained on medical therapy indefinitely.

##### Hairline lowering surgery

This can be considered in patients with FFA if the disease is inactive. It is optimally timed following >24 months of inactivity. There was a CD for inactivity of 6 months duration.

##### Micro needling

There was no expert consensus in this category.

#### Laser and light

There was NC for any questions regarding laser and light therapy in FFA. AC: Fractional ablative CO_2_ laser is not an effective treatment option for FFA*.

### Usefulness of therapy

In this section, experts selected useful therapies in FFA (Table 2). Expert consensus was reached in 26 of 55 questions (47%).

**TABLE 2:** Expert consensus agreement for useful FFA treatment.

Category	Useful	Not useful
Topical therapy	Topical corticosteroids Topical tacrolimus Topical minoxidil	Topical 1% dithranol (anthralin) Topical antihistamines Topical tretinoin
Intralesional therapy	Intralesional corticosteroid injections as monotherapy Intralesional corticosteroid injections combined with topical minoxidil Intralesional corticosteroid injections combined with oral agent	Interferon‐alpha
Systemic therapy	Hydroxychloroquine Tetracyclines (lymecycline, doxycycline, minocycline) Systemic corticosteroids Ciclosporin Methotrexate Finasteride Dutasteride Isotretinoin Oral minoxidil	Oral antihistamines
Physical therapy	Hair transplantation	Fractional ablative CO2 laser Fractional non‐ablative laser Micro‐needling

### Cosmetic camouflage and counselling

Eight questions featured in this category. Expert consensus was reached in 5 (63%).

Cosmetic camouflage should be discussed with all patients. Patients should be counselled not to avoid using sunscreen completely and not to selectively avoid chemical or physical sunscreens. Counselling advice to use alternative modes of sun protection, for example, hat. AC: to avoid sunscreen use on the forehead/ eyebrow region**.

### Treatment initiation and potential discontinuation

Fifty‐six questions related to this category. Expert consensus was reached in 34 (61%).

Objective monitoring is appropriate for asymptomatic patients with a good history of stability.

Consider treatment at the time of diagnosis, even if signs of clinical inflammation are absent. FFA treatment regimen to include topical and systemic therapy if no contraindications (Table 2), AC: not necessarily routinely include an intralesional agent**. FFA patients require aggressive (i.e. systemic) treatment, but this should be dependent on, and tailored to, patient factors (expectations of impact on QoL).

Active treatment is expected to improve patient outcomes. In general, combination treatment is more effective than monotherapy.

Treatment should be trialled for 6 months (AC up to 12 months**; CD 3, 18, 24, >24 months) before determining effectiveness (i.e. if ineffective at that stage consider cessation).

Treatment discontinuation may be considered where FFA is inactive after 24 months (CD 3, 6 months or never) with 5ARI therapy (finasteride/dutasteride), 12 months (CD 3, 6, 18, 24 months or never) with anti‐malarial therapy (hydroxychloroquine), 6 months (CD 3, 24 months or never) with ciclosporin, 6 months (CD 3 months or never) with retinoids.

While it was agreed that treatment with MTX or MMF should not be continued indefinitely, there was no agreement for the specific timing of treatment discontinuation in women with inactive FFA. However, there was CD regarding stopping MTX/MMF prematurely, that is, after only 3 months of disease inactivity (inactivity for this period indicating disease remission.) and discontinuing MMF after prolonged disease inactivity, that is, after 2 years.

### Disease monitoring

Nine questions featured in this category. Expert consensus was reached in all 9 (100%).

Acceptable objective measures of improvement include the following: Frontal Fibrosing Alopecia Severity Index FFASI,^17^ Frontal Fibrosing Alopecia Severity Score FFASS,^18^ serial measurement of the frontal hairline,^3^, ^19^ serial photography, trichoscopy^20^ and video‐trichoscopy.

Reliable measures of ongoing activity include the presence of symptoms, clinical inflammation (e.g. peripilar scaling/erythema) and a positive anagen hair pull test.

### Prognostic factors and natural disease course

Eighteen questions featured in this category. Expert consensus was reached in 12 (67%).

Factors affecting prognosis include the presence of symptoms, clinical inflammation (peripilar scaling/erythema), a positive anagen hair pull test, facial papules, non‐scalp involvement (axillary, pubic and body hair loss [The authors acknowledge that axillary and pubic hair loss can also occur with physiological ageing]), diffuse FFA pattern and pseudo‐ophiasis FFA pattern.

#### Disease course

The tendency is for slow progression then spontaneous stabilization. Disease reactivation may occur after treatment withdrawal following a period of stabilization. Treatment has the potential to achieve hair regrowth on the eyebrows. AC: treatment has the potential to achieve regrowth on the scalp **.

## PREFERRED TREATMENT

Experts were asked to list in order their choice of first, second‐ and third‐line treatment for FFA.

**FIGURE 3:**
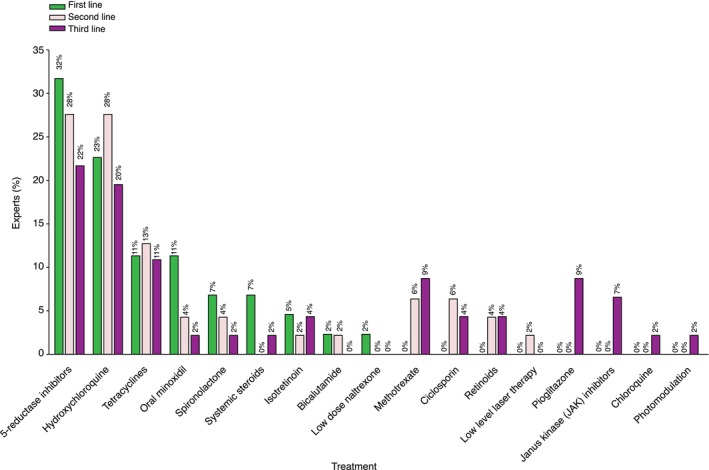
Expert opinion: First‐, second‐ and third‐line systemic treatment for FFA.

The most common systemic agent utilized by the expert group was 5ARIs followed by hydroxychloroquine (Figure 3). The most frequent topical/physical modality used as first‐line treatment, alone or in combination with a systemic agent, was topical corticosteroids followed closely by intralesional corticosteroids (Figure S1).

### FFA eyebrow alopecia

Intralesional corticosteroids were most commonly reported as the preferred treatment for FFA‐induced eyebrow alopecia, followed by topical calcineurin inhibitors (Figure S2).

### FFA facial papules

Oral isotretinoin was most commonly reported as the preferred treatment for FFA‐induced facial papules, followed by topical retinoids (Figure S3).

## DISCUSSION

SOFFIA, one of the largest eDelphi consensus exercises to date, explores diverse FFA treatment options. The goal of management in FFA is to arrest disease progression, reduce inflammation, control symptoms and manage expectations. The permanent hair loss with FFA prompted consensus that topical and systemic therapy is commenced early, even in the absence of overt clinical inflammation and in seemingly stable patients. Although hair regrowth can occur in exceptional cases, especially if treatment is initiated early, the expert group concurred that hair growth is typically not to be expected.^21^

5ARIs were considered effective for FFA (finasteride 2.5 daily or dutasteride 0.5 mg daily) and the preferred first‐line systemic treatment. This corroborates with existing studies demonstrating effectiveness and disease stabilization as mono/combination therapy.^22^, ^23^ While limited data raised breast cancer risk concern,^24^ a recent large‐scale, confounder‐adjusted study of 1000 patients confirmed no evidence of an association between finasteride use and male breast cancer.^25^

Low‐dose oral minoxidil (LDOM) is increasingly being used in many alopecias at doses up to 2.5 mg/day, and consensus agreement was reached for LDOM as an adjunct in FFA and a useful therapy (Table 2). Although the exact mechanism is incompletely understood, reversal of miniaturization with anagen prolongation, optimizing overall hair density, may contribute in FFA. This is particularly true for FFA patients with co‐existing androgenetic alopecia.

Systemic corticosteroids were considered an effective treatment in rapidly progressive frontal fibrosing alopecia (RPFFA). While considered to be useful (Table 2) in unstable inflammatory disease, optimal dosing was not agreed upon, though there was agreement that high‐dose therapy (1 mg/kg/day) and very low‐dose therapy (<0.25 mg/kg/day) were both inappropriate. The consensus trend was towards 0.5 mg/kg/day, gradually tapered. Importantly, experts deemed this to be a short‐term measure, not a long‐standing maintenance treatment.

Hair transplantation suitability is of significant interest to FFA patients. Acknowledging limited data, the group agreed that with careful selection, counselling, disease stability for at least 2 years and continuation of medical therapy to prevent koebnerization/disease reactivation, transplantation may be an option.^26^

With up to 90% of FFA patients reporting eyebrow loss, preceding scalp alopecia in 39% of cases, site‐specific treatment is important.^17^, ^22^, ^27^ Consensus agreement and limited data suggest regrowth potential with some therapies.^15^, ^28^, ^29^ Intralesional corticosteroids were the most frequent expert‐selected therapy for this site (36; 29%); administered as intralesional triamcinolone 2.5 mg/mL every 6–8 weeks (Figure S2). Topical calcineurin inhibitors were also considered effective (23;18%).

In line with SOFFIA findings, low‐dose oral isotretinoin has been shown to improve facial papules in FFA and may also benefit the hairline.^30^

The use of validated measures of disease activity, such as the FFASI^17^ and FFASS,^18^ was recommended, though it was acknowledged practicality may preclude use in all clinical settings. Serial photography and serial measurements^3^, ^19^ were also considered reliable methods of disease monitoring.

SOFFIA findings indicate that cautious attempts to cease systemic therapy can be made if disease remission is confidently established or if there are drug safety concerns prohibiting long‐term use. Longer durations of disease inactivity were recommended for 5ARIs and antimalarial therapy, but nephrotoxicity and hypertension should limit ciclosporin treatment duration. Limited data likely contributed to a lack of consensus regarding JAKi (e.g. tofacitinib, ruxolitinib) therapy in FFA.

Though a strength of the study was global representation, limited availability of therapies in some countries (e.g. lymecycline is unavailable in Australia) prohibited voting by all members for certain therapies. Given the COVID‐19 restrictions, forty‐two (61%) experts participated in the final stage of the Delphi process. Further limitations included disproportionately low representation from South America (3%) and Africa (5%), restricted capacity to assess all therapies in detail, for example, apremilast,^31^ not capturing experts' years of clinical practice, the effect of COVID‐19 on data collection and consensus threshold of ≥66%. While quality treatment data are currently lacking (Table S1), SOFFIA identifies the pressing need for more robust evidence‐based treatments for FFA.

## CONCLUSIONS

SOFFIA provides a unique opportunity to draw on the expertise of geographically disparate clinicians; minimising peer influence to provide consensus, where possible, on contentious/debated treatments in FFA, while acknowledging that local factors, for example, availability of services will ultimately impact decision‐making. Overall, topical steroids were the preferred first‐line topical therapy. Preferred systemic treatments were 5ARIs, followed by hydroxychloroquine. Multiple factors can affect FFA prognosis, including the presence of symptoms, facial papules and non‐scalp involvement.

**FIGURE 4:**
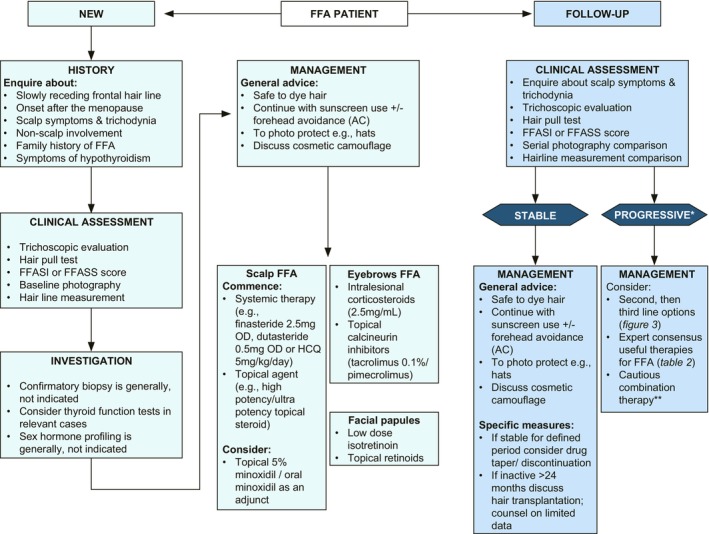
SOFFIA: consensus assessment and management algorithm. AC, approaching consensus; FFA, frontal fibrosing alopecia; FFASI/FFASS, frontal fibrosing alopecia severity index/scale; HCQ, hydroxychloroquine; OD, once daily; SOFFIA, statement from the frontal fibrosing alopecia international expert alliance.*Despite dose optimization. **Note for contraindications/interactions.

The findings from SOFFIA provide a framework for the development of local guidelines for FFA. Figure 4 is a summary of the consensus points on management.

## AUTHOR CONTRIBUTIONS

Nekma Meah: conceptualization, data acquisition, analysis, drafting the article for intellectual content, approval of final manuscript. Jane Li: conceptualization, data acquisition, analysis, drafting the article for intellectual content, approval of final manuscript. Dmitri Wall: conceptualization, data acquisition, analysis, drafting and critical review of the article for intellectual content, approval of the final manuscript. Katherine York: conceptualization, data acquisition, analysis, drafting and critical review of the article for intellectual content, approval of final manuscript. Bevin Bhoyrul: conceptualization, data acquisition, analysis, drafting and critical review of the article for intellectual content, approval of final manuscript. Laita Bokhari: conceptualization, data acquisition, analysis, critical review of the article for intellectual content, approval of final manuscript. Lachlan Coulthard: conceptualization, data acquisition, analysis, critical review of the article for intellectual content, approval of final manuscript. Leila Asfour: data acquisition, analysis, critical review of the article for intellectual content, approval of final manuscript. Leonardo Spagnol Abraham: data acquisition, critical review of the article for intellectual content, approval of final manuscript. Daniel Asz‐Sigall: data acquisition, critical review of the article for intellectual content, approval of final manuscript. Wilma F Bergfeld: data acquisition, critical review of the article for intellectual content, approval of final manuscript. Regina C. Betz: data acquisition, critical review of the article for intellectual content, approval of final manuscript. Ulrike Blume‐Peytavi: data acquisition, critical review of the article for intellectual content, approval of final manuscript. Valerie Callender: data acquisition, critical review of the article for intellectual content, approval of final manuscript. Vijaya Chitreddy: data acquisition, critical review of the article for intellectual content, approval of final manuscript. Andrea Combalia: data acquisition, critical review of the article for intellectual content, approval of final manuscript. George Cotsarelis: data acquisition, critical review of the article for intellectual content, approval of final manuscript. Brittany Craiglow: data acquisition, critical review of the article for intellectual content, approval of final manuscript. Rachita Dhurat: data acquisition, critical review of the article for intellectual content, approval of final manuscript. Ncoza Dlova: data acquisition, critical review of the article for intellectual content, approval of final manuscript. Jeff Donovan: data acquisition, critical review of the article for intellectual content, approval of final manuscript. Andrei Doroshkevich: data acquisition, critical review of the article for intellectual content, approval of final manuscript. Samantha Eisman: data acquisition, critical review of the article for intellectual content, approval of final manuscript. Paul Farrant: data acquisition, critical review of the article for intellectual content, approval of final manuscript. Aida Gadzhigoroeva: data acquisition, critical review of the article for intellectual content, approval of the final manuscript. Jack Green: data acquisition, critical review of the article for intellectual content, approval of final manuscript. Ramon Grimalt: data acquisition, critical review of the article for intellectual content, approval of final manuscript. Matthew Harries: data acquisition, critical review of the article for intellectual content, approval of final manuscript. Maria Hordinsky: data acquisition, critical review of the article for intellectual content, approval of final manuscript. Alan D. Irvine: data acquisition, critical review of the article for intellectual content, approval of final manuscript. Victoria Jolliffe: data acquisition, critical review of the article for intellectual content, approval of final manuscript. Spartak Kaiumov: data acquisition, critical review of the article for intellectual content, approval of final manuscript. Brett King: data acquisition, critical review of the article for intellectual content, approval of final manuscript. Steven Kossard: data acquisition, critical review of the article for intellectual content, approval of final manuscript. Joyce Lee: data acquisition, critical review of the article for intellectual content, approval of final manuscript. Won‐Soo Lee: data acquisition, critical review of the article for intellectual content, approval of final manuscript. Nino Lortkipanidze: data acquisition, critical review of the article for intellectual content, approval of final manuscript. Amy McMichael: data acquisition, critical review of the article for intellectual content, approval of final manuscript. Natasha Atanaskova Mesinkovska: data acquisition, critical review of the article for intellectual content, approval of final manuscript. Andrew Messenger: data acquisition, critical review of the article for intellectual content, approval of final manuscript. Paradi Mirmirani, data acquisition, critical review of the article for intellectual content, approval of final manuscript. Elise Olsen: data acquisition, critical review of the article for intellectual content, approval of final manuscript. Seth J. Orlow: data acquisition, critical review of the article for intellectual content, approval of final manuscript. Yuliya Ovcharenko: data acquisition, critical review of the article for intellectual content, approval of final manuscript. Bianca Maria Piraccini: data acquisition, critical review of the article for intellectual content, approval of final manuscript. Rodrigo Pirmez: data acquisition, critical review of the article for intellectual content, approval of final manuscript. Adriana Rakowska: data acquisition, critical review of the article for intellectual content, approval of final manuscript. Pascal Reygagne: data acquisition, critical review of the article for intellectual content, approval of final manuscript. Janet Roberts: data acquisition, critical review of the article for intellectual content, approval of final manuscript. Lidia Rudnicka: data acquisition, critical review of the article for intellectual content, approval of final manuscript. David Saceda‐Corralo: data acquisition, critical review of the article for intellectual content, approval of final manuscript. Jerry Shapiro: data acquisition, critical review of the article for intellectual content, approval of final manuscript. Pooja Sharma: data acquisition, critical review of the article for intellectual content, approval of final manuscript. Tatiana Silyuk: data acquisition, critical review of the article for intellectual content, approval of final manuscript. Poonkiat Suchonwanit: data acquisition, critical review of the article for intellectual content, approval of final manuscript. Anita Takwale: data acquisition, critical review of the article for intellectual content, approval of final manuscript. Antonella Tosti: data acquisition, critical review of the article for intellectual content, approval of final manuscript. W I Visser: data acquisition, critical review of the article for intellectual content, approval of final manuscript. Sergio Vañó‐Galván: data acquisition, critical review of the article for intellectual content, approval of final manuscript. Annika Vogt: data acquisition, critical review of the article for intellectual content, approval of final manuscript. Martin Wade: data acquisition, critical review of the article for intellectual content, approval of final manuscript. Leona Yip: data acquisition, critical review of the article for intellectual content, approval of final manuscript. Abraham Zlotogorski: data acquisition, critical review of the article for intellectual content, approval of the final manuscript. Cheng Zhou: data acquisition, critical review of the article for intellectual content, approval of final manuscript. Rodney Sinclair: conceptualization, data acquisition, critical review of the article for intellectual content, approval of the final manuscript, supervision.

## FUNDING INFORMATION

National and International Skin Registry solutions (NISR) for facilitating videoconferencing facilities. Australasian Hair and Wool Research Society for funding of the eDelphi software. MH is supported by the NIHR Manchester Biomedical Research Centre (NIHR203308).

## CONFLICT OF INTEREST STATEMENT

Nekma Meah: Eli‐Lilly –Honoraria, Dermal—Honoraria, Pfizer—Honoraria/Consultancy/Advisory Board. Sunpharm (Advisory Board). Dmitri Wall: Has received honoraria for consultancy from Pfizer, Eli‐Lillyand Bristol Myers Squibb, has received speaker fees from L'Oreal and Almirall, and received support from AbbVie to attend conferences. He has consulted for Sun Pharma. He is a shareholder in Samson Clinical. He is an employee of National and International Skin Registry Solutions (NISR) and a director of Hair Restoration Blackrock. He is a Steering Committee member of the Global Registry of Alopecia Areata Disease Severity and Treatment Safety (GRASS) International and PI of GRASS Ireland. Bevin Bhoyrul: Advisory board for L'Oréal Australia. Principal investigator: Amgen. Laita Bokhari, Lachlan Coulthard, Leila Asfour: none. Leonardo Spagnol Abraham: Vichy Laboratoires and GSK—speaker; Pfizer—speaker/advisory board; –investigator—Johnson & Johnson; CEO–Trichology Club. Daniel Asz‐Sigall: none. Wilma F Bergfeld, Lilly (investigator); Pfizer (investigator); AbbVie (investigator). Regina C. Betz: a consultant for Pfizer. Ulrike Blume‐Peytavi: a speaker and/or consultant and/or investigator and/or has received research funding from AbbVie, Adaran, Allmiral, Bayer Health Care, Ameryt, Boots Healthcare, Cantabria Labs, Cassiopeia, CeraVe, Dermocosmétique Vichy, Eli Lilly, FomF, Galderma Laboratorium GmbH, GSK, Infectopharm, Johnson & Johnson, Laboratoires Bailleuil, Legacy, Leo Pharma, Novartis, Pfizer, Pierre Fabre, Sanofi Regeneron and Sun Pharmaceutical. Valerie Callender: Acne store (Consultant), Abbvie/Allergen (Researcher), Aerolase (Consultant), Arcutis (Consultant/Speaker), Avava (Researcher), Avita medical (Consultant) Beiersdorf (Consultant), Cutera (consultant), Dermavant (Consultant), Eli Lilly (Consultant/Researcher/Speaker), Galderma (Researcher), Incyte (consultant/Researcher), Juenes Aesthetics (Consultant), Loreal Aesthetics (Consultant/Researcher), Ortho Derm (Consultant), I Know Skincare (Researcher), Janssen (Researcher), Pfizer (Researcher/Consultant), Prollineum (Researcher), Skinbetter Science(researcher), Skin Ceuticals (consultant), Symatèse (Researcher), Teoxane (Researcher), Uptodate(Royalties). Vijaya Chitreddy: none. Andrea Combalia: speaker and/or consultant and/or investigator and/or has received research funding from Lilly, Pfizer, Takeda, Kyowa. George Cotsarelis: none. Brittany Craiglow (BC): has received honoraria and/or fees from AbbVie, Arcutis, BiologicsMD, Dermavant, Incyte, Eli Lilly, LEO Pharma, Glaxo Smith Klein, Novartis, Pfizer, Regeneron, Sanofi‐Genzyme and Sun Pharmaceuticals. Rachita Dhurat, Ncoza Dlova: none. Jeff Donovan: Honoraria—Pfizer Vichy. Royalties—Up to Date. Andrei Doroshkevich: none. Samantha Eisman: investigator in clinical trials and/or affiliated with (Presentations/Promotional work/Advisory boards) for AbbVie, Arena Pharmaceutical, Boston Pharmaceuticals, Botanix, Bristol‐Myers Squibb, Celldex Therapeutics, Dermaliq Therapeutics, Dermira, Eli Lilly and Company, Evelo Biosciences, Hope Medicine, Immunic Therapeutics, Janssen, Kobiolabs, Kymab, LEO Pharma, L'oreal, Nektar Therapeutics, Novartis, Pfizer Inc., Regeneron, Sanofi, Suzhou Connect Biopharmaceuticals, Takeda Pharmaceuticals, TEVA Pharmaceuticals, Tigermed and Zai Lab. I am the treasurer and board member of the Australian Hair and Wool Research Society (2019 to present). Paul Farrant: principal investigator for Pfizer, AbbVie, Soterios in commercial alopecia areata research and paid speaker for Pfizer and Vichy. Aida Gadzhigoroeva, Ramon Grimalt: none. Matthew Harries: served as a speaker for Pfizer (paid to institution); the principal investigator on the Soterios (Manentia UK) clinical trial for alopecia areata, the AA‐UP (AbbVie) clinical trial and an alopecia areata clinical trial (Sanofi); has held consultancy roles for AbbVie, Eli Lilly and Pfizer (paid to his institution); has served as an expert member/contributor for the National Institute for Health and Care Excellence; has received grants from Alopecia UK, British Skin Foundation and UCB; is the programme lead for the NIHR Manchester Biomedical Research Centre Inflammatory Hair Diseases and the British Association of Dermatologists Alopecia Areata National Guideline 2024; and is supported by the NIHR Manchester Biomedical Research Centre (NIHR203308). Maria Hordinsky: Board of Directors: American Academy of Dermatology and the scarring Alopecia Foundation (unpaid). Alan D. Irvine: Fees from AbbVie, Eli Lilly, Pfizer, Benevolent AI, Arena, Novartis, Regeneron, Sanofi, Leo Pharma, Janssen, OM Pharma, Moon lake and Almirall. President of international Eczema Council. Victoria Jolliffe, Steven Kossard, Joyce Lee, Won‐Soo Lee, Nino Lortkipanidze: none. Amy McMichael: Grants/Research: Concert, Procter and Gamble, Incyte and Revian. Consulting: Lilly, Janssen, Pfizer, Arcutis, Almirall, AbbVie, Cerave, Galderma, Bristol Meyers Squibb, Sanofi Regeneron, Sun Pharma, UCB, Pelage, Procter and Gamble, Revian, Johnson & Johnson, L'oreal and Leo. Royalties: Informa/Taylor and Francis and McGraw Hill. Natasha Atanaskova Mesinkovska: Consultant/Honoraria: AbbVie, Biologics MD, Lilly, Pfizer, Merck, Sun Pharma, Loreal, Nutrafol. Speaker: Lilly and Pfizer. Andrew Messenger: Samson Clinical. Paradi Mirmirani: Amgen (Investigator)—Grants/Research Grants. Cicatricial Alopecia Research Foundation (Advisory Board)—no compensation. Eli Lilly and Company—Investigator (Grants/Research Grants). Incyte Corporation—Investigator—(Grants/Research Grants). National Alopecia Areata Foundation—Clinical Research Advisory Council—No Compensation. NeoGenesis—Consultant—Fees. Pfizer—Grants/Research Grants/ Advisory Board. Springer Science—Royalties. Sun Pharmaceuticals (Concert Pharmaceuticals)—Grants/Research Grants/ Advisory Board. UpToDate—Royalties. Elise Olsen: Eli Lilly (consulting fees); Scarring Alopecia Foundation BOD (unpaid). Seth J. Orlow: I serve on the Board of Almirall Srl and R2 Technologies and as an Advisor to Evommune and Pharus Advisors. Yuliya Ovcharenko: speaker and/or consultant and/or investigator for Vichy Dercos, Pfizer, FotoFinder and Tricopat. Bianca Maria Piraccini: Pierre Fabre Ducray (Consultant/Honoraria), Difa Cooper Cantabria. Labs (Consultant/Honoraria), Dercos‐L'Oreal Paris (Consultant/Honoraria), ISDIN (Consultant/Honoraria), Legacy Healthcare (Consultant/Honoraria), Eli Lilly(Consultant/Honoraria), Pfizer (Consultant/Honoraria). Dr. Rodrigo Pirmez received fees from Aché, Pfizer, Eli Lilly, Darrow, Vichy, GlaxoSmithKline and Tricholab. Adriana Rakowska: none. Pascal Reygagne: speaker or investigator or member of a board for AbbVie, BMS, Cosmétique active, Ducray, Legacy Healthcare, Lilly, L'Oréal recherche, Pfizer, Pierre Fabre Dermatologie, Sun Pharma and Vichy. Janet Roberts: none. Lidia Rudnicka: invited medical lectures for Leo Pharma, Pfizer, L'Oreal, Sanofi and Eli Lilly; advisory board: Leo Pharma, Janssen Pharmaceutical Companies, Novartis, Pfizer and UCB. David Saceda‐Corralo: COI investigator in clinical trials and/or affiliated with (presentations/advisory boards) for Eli Lilly and Company, L'Oreal, Pfizer Inc., ISDIN, Cantabria Labs. Vice president of the Spanish Hair Research Society (2023 to present). Jerry Shapiro: Pfizer (investigator and consultant). Lilly (consultant and medical advisory board). Pooja Sharma, Tatiana Silyuk, Poonkiat Suchonwanit: none. Anita Takwale: educational speaker fees from Pfizer, Sanofi, L'Oreal and Vichy. Antonella Tosti: Consultant DS Laboratories, Almirall, Tirthy Madison, Eli Lilly, Pfizer, Myovant, Bristol Myers Squibb, Ortho Dermatologics and Sun Pharmaceuticals. W I Visser: received honoraria from Pfizer, Eli Lilly, AbbVie, Novartis, Sanofi and Galderma. Involved in clinical trials with AbbVie. Sergio Vañó‐Galván: AbbVie Speaker Fees; Cantabria Labs Advisory Board Fees; Fotofinder Speaker Fees; Lilly ICOS LLC Advisory Board Fees; L'Oréal France Advisory Board Fees; Pfizer Inc. Advisory Board Fees; Pierre Fabre Dermatologie Speaker Fees. Chair or Member of a Council, Committee, Task Force, Ad Hoc Task Force or Work Group; Candidate for Office or am a member of the Board of Directors; Editor of the Journal of the American Academy of Dermatology or Dermatology World. Annika Vogt: Honoraria Bayer, Derma@Home, Infectopharm, Sanofi, UCB Pharma GmbH, Pfizer, Amryt. Board Member Work Group for Pediatric Dermatology, Chair, Vice‐Chair Position. Martin Wade: None. Leona Yip: KOL/consultant for L'Oreal, Galderma, Leo Pharma, Eli Lilly, Pfizer. Abraham Zlotogorski: Investigator for Eli Lilly and Company, and consultant for Pfizer. Rodney Sinclair: Pharmaceutical advisory board Eli Lilly and Company, Pfizer Inc., Leo Pharmaceutical, AbbVie. Speaker bureau Pfizer, AbbVie, Novartis. Glaxo Smith Kline, Eli Lilly. Principal investigator in clinical trials for AbbVie, Aerotech, Akesobio, Amgen, Arcutis, Arena, Ascend, AstraZeneca, Bayer AG, Biotherapeutics, Boehringer Ingelheim, Bristol Myer Squibb, Celgene, Coherus BioSciences, Connect, Demira, Dermaliq, Eli Lilly and Company, Galderma, Glaxo Smith Kline, F. Hoffman–La Roche, Incyte, Janssen, MedImmune, Merck and Co, Merck Sharpe & Dohme, Novartis, Oncobiologics, Pfizer, Principia, Regeneron, Roche, Reistone Biopharma, Samson Clinical, Sanofi‐Genzyme, Samson Clinical, Sun Pharma, UCB, Valeant and Zai Labs. President of the Australasian Hair and Wool Research Society, Vice President of the International Society of Dermatology, Vice President of The International Academy of Dermatology. Board member Australian Society of Dermatology research. Assistant Editor, Australian Journal of Dermatology. Dr. Sinclair is the Director and Founder of Samson Medical Pty Ltd. which holds patents on the use of oral minoxidil to treat hair loss disorders; is on the Pharmaceutical advisory board of Eli Lilly, Pfizer Inc. and Leo Pharmaceutical; is on the Speaker bureau of AbbVie, Novartis, Glaxo Smith Kline; is a principal investigator in clinical trials for Amgen, Novartis, Arcutis Biotherapeutics, Aerotech, Merck and Co, Celgene, Coherus BioSciences, Janssen, Regeneron, MedImmune, Glaxo Smith Kline, Samson Clinical, Boehringer Ingelheim, Oncobiologics, Roche, Ascend, Dermira, AstraZeneca, Akesobio, Reistone Biopharma, UCB Biopharma, Sanofi, Connect, Arena, Sun Pharma, Bristol Myer Squibb, Botanix Pharmaceuticals, Pfizer, Eli Lilly, Zai Pharmaceuticals, Jiangsu Hengrui Medicine, Leo Pharma, AbbVie, AstraZeneca and Galderma. The other authors declare no conflicts of interest.

## ETHICAL APPROVAL

Not applicable.

## ETHICS STATEMENT

Written informed patient consent has been obtained for the clinical image for SOFFIA. We are committed to conducting research with integrity, transparency and respect for all participants, following the principles outlined in the Declaration of Helsinki and applicable ethical guidelines. The results presented are aimed at contributing to the broader understanding of the topic, without compromising the rights and welfare of any individuals.

## Supporting information


Table S1.

Table S2.

Figure S1.

Figure S2.

Figure S3.

## Data Availability

The data that support the findings of this study are available from the corresponding author upon reasonable request.
