# Supplementary material for: Statement from the frontal fibrosing alopecia international expert alliance: SOFFIA 2024
Source: J Eur Acad Dermatol Venereol. 2025 Jul 23;40(2):210–23. doi: 10.1111/jdv.20833 (PMC12843854; doi:10.1111/jdv.20833)
Supplement: Supplementary file 1 — Table S1. [file JDV-40-210-s005.docx]

Table 1: Level of evidence for FFA treatments based on the OCEBM criteria

| Treatment | Level of Evidence |
| --- | --- |
| Topical corticosteroids | II |
| Topical tacrolimus | II |
| Topical minoxidil | III |
| Topical JAK inhibitors | II |
| Topical dithranol | IV |
| Topical antihistamines | IV |
| Topical tretinoin | IV |
| Intralesional corticosteroids | III |
| Intralesional corticosteroids in combination with topical minoxidil | III-IV |
| Intralesional corticosteroids in combination with oral agent | III-IV |
| Hydroxychloroquine | II |
| Tetracyclines | III |
| Systemic corticosteroids | III |
| Ciclosporin | IV |
| Methotrexate | III |
| Finasteride | II |
| Dutasteride | III |
| Isotretinoin | III |
| Oral minoxidil | III |
| Interferon alpha | IV |
| Oral JAK inhibitors | IV |
| Oral antihistamines | IV |
| Hair transplantation | IV |
| Fractional ablative CO2 | IV |
| Fractional non-ablative laser | IV |
| Micro-needling | IV |
