# Supplementary figures and images for: Statement from the frontal fibrosing alopecia international expert alliance: SOFFIA 2024
Source: J Eur Acad Dermatol Venereol. 2025 Jul 23;40(2):210–23. doi: 10.1111/jdv.20833 (PMC12843854; doi:10.1111/jdv.20833)

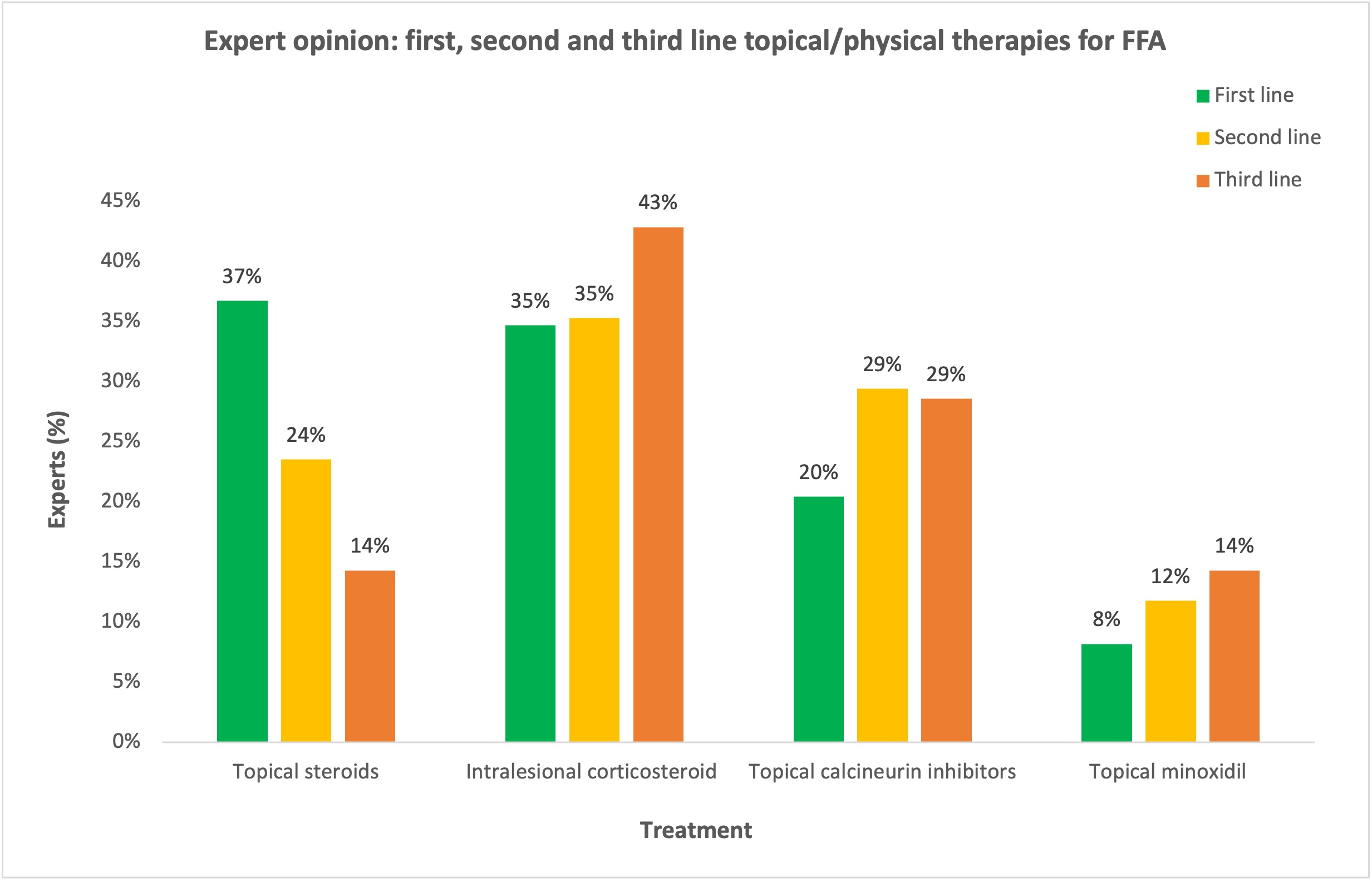

Supplement: Supplementary file 3 — Figure S1. [file JDV-40-210-s002.jpg]

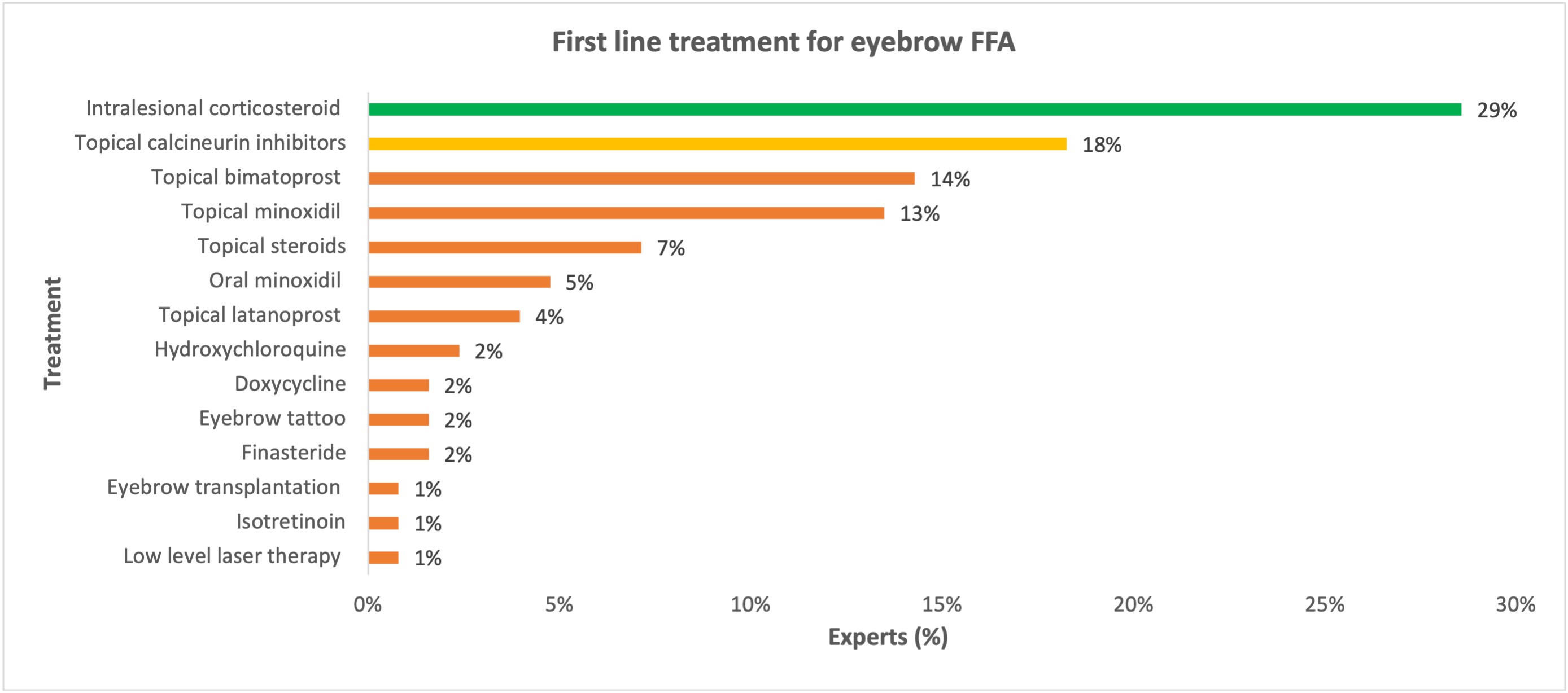

Supplement: Supplementary file 4 — Figure S2. [file JDV-40-210-s001.jpg]

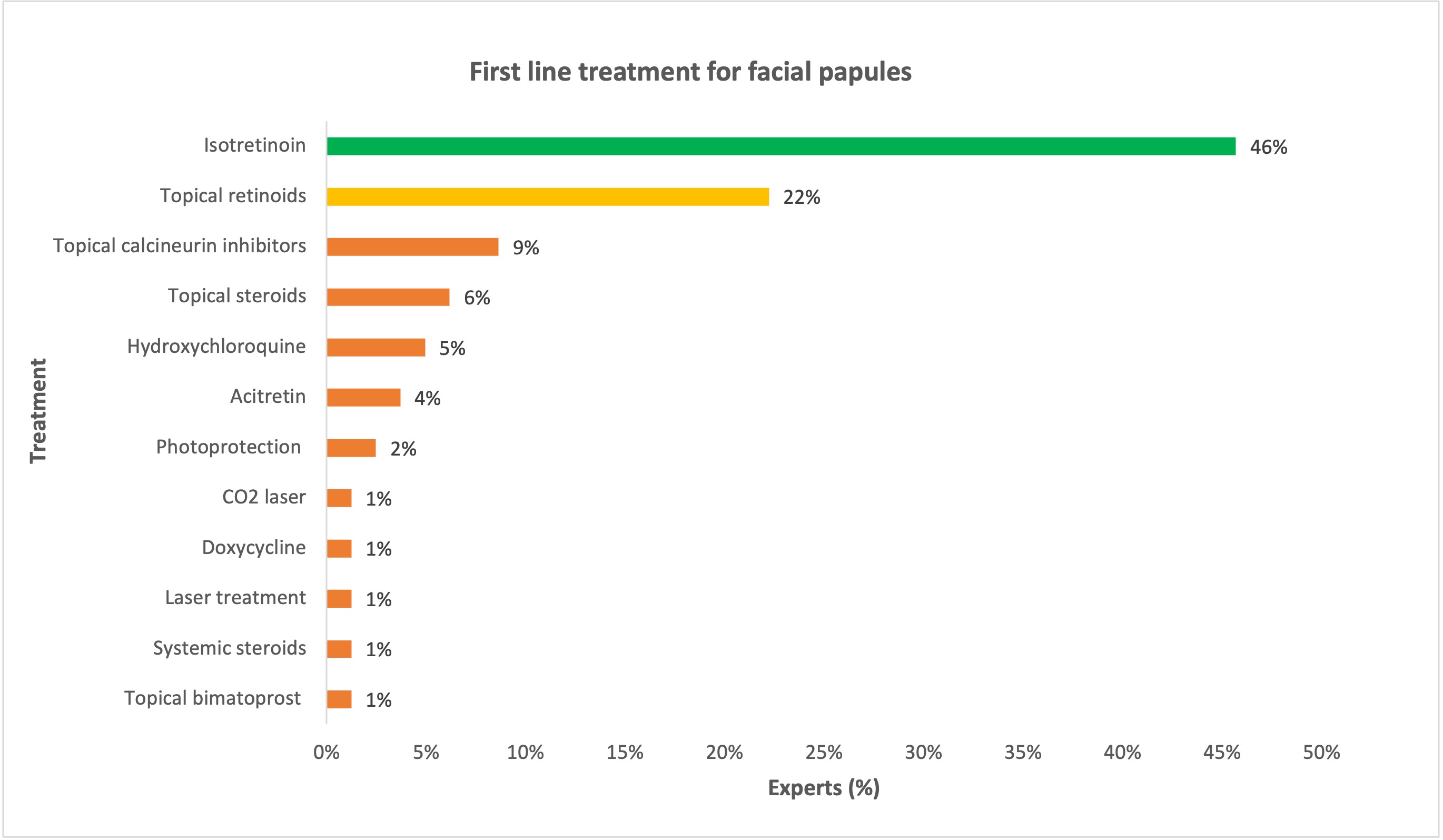

Supplement: Supplementary file 5 — Figure S3. [file JDV-40-210-s004.jpg]
